# Involuntary Smoking in Adolescents, Their Awareness of Its Harmfulness, and Attitudes towards Smoking in the Presence of Non-Smokers

**DOI:** 10.3390/ijerph14101095

**Published:** 2017-09-21

**Authors:** Dorota Kaleta, Kinga Polanska, Piotr Wojtysiak, Franciszek Szatko

**Affiliations:** Department of Hygiene and Epidemiology, Medical University of Lodz, 90-647 Lodz, Poland; kinga.polanska@umed.lodz.pl (K.P.); pwojtysiak@wp.pl (P.W.); franciszek.szatko@umed.lodz.pl (F.S.)

**Keywords:** youth, adolescents, involuntary smoking, passive smoking, prevalence, beliefs and attitudes towards exposure, tobacco control, public health

## Abstract

The aim of the study was to examine involuntary smoking among young people, their awareness of its harmfulness and the factors associated with attitudes towards smoking in the presence of non-smokers. A cross-sectional study was conducted among 3552 students from a socially disadvantaged rural area in central Poland. Almost 40% of the participants were exposed to involuntary smoking at home and 60% outside of home on a daily or almost daily basis. More than 80% of the students felt that smoking should be banned around children at home, 59% thought it should be banned in vehicles, and 41% in the presence of non-smokers. The majority of the students were aware of the health consequences of active smoking, and 69% understood the threats of passive smoking. Females, never-smokers and current non-smokers, as well as those without smoking parents were more likely to claim that smoking should be banned at home and in vehicles (*p* < 0.05). Those aware of the fact that smoking was harmful to health, who discussed those issues with their parents and teachers, and who saw school tobacco control policies, were more likely to maintain that passive smoking should be banned (*p* < 0.05). The study results highlight the need for programs and policies to eliminate involuntary smoking among young people.

## 1. Introduction

Mainstream smoke inhaled by a smoker contains over 4000 chemicals, including irritants and almost 70 carcinogens [1,2,3]. Involuntary smoking, also known as passive smoking, second-hand smoke (SHS) or environmental tobacco smoke (ETS) exposure, refers to the mixture of side stream smoke coming from the burning tip of a cigarette and exhaled mainstream smoke. The concentration of toxins and carcinogens in side stream smoke is often much higher than it is in mainstream smoke. Based on the existing evidence, there is no safe level of involuntary smoking [1,2,3,4]. In addition, considering their developing body as well as a higher level and longer time of exposure, children and adolescents are at a greater risk of future health consequences of the exposure [3]. These include upper and lower respiratory tract infections, decreased lung function, asthma, middle ear diseases and neurodevelopmental outcomes, such as behavioral problems or decreased cognitive and psychomotor functions. Passive smokers are also more likely to initiate and continue the use of cigarettes as compared to those who are prevented from ETS exposure [3,5].

While the adoption of comprehensive legislation in most countries which have ratified the World Health Organization (WHO) Framework Convention on Tobacco Control has led to a significant decline in tobacco smoke exposure, the legislation in many cases does not cover environments where young people spend much of their time, or where the exposure is higher, i.e., at home and/or in vehicles [6,7]. Based on the WHO estimates, 40% of the world’s children are still exposed to passive smoking at home [8]. An analysis among never-smoking adolescents in 168 countries has indicated that 30% of them were exposed to involuntary smoking at home, 44% outside of it, and 23% in both places [9]. It needs pointing out that the largest proportion of these young people lived in the European region (54%, 74%, and 46%, respectively). The interior of a vehicle is another common and a particularly dangerous source of exposure for the youth. Involuntary smoking in vehicle, due to the small enclosed space, is even up to 23 times more toxic than the exposure occurring at home [10,11,12].

An analysis performed among Canadian adolescents has indicated that the majority of them did not think that smoking should be allowed around children at home or in a vehicle [10,11,12]. It has been also observed that males, never smokers and young people living in a house where someone smokes were all associated with being more likely to claim that smoking around adolescents should be banned.

The implementation of effective tobacco control measures requires a better understanding of the factors associated with the beliefs the youth have about involuntary smoking. It can be expected that young people’s perception of the issue may be country-specific and can depend on the level of exposure on the one hand, and on the existence and awareness of tobacco control policies on the other.

Despite the fact that tobacco control activities and enforcement of the legislation are relatively well developed, these are mostly carried out among adults in metropolitan areas [13,14]. Remote rural regions and disadvantaged populations, which are more difficult to reach, are less frequently covered by these activities [15,16,17]. Even though Poland has implemented legal limitations on smoking and a complete ban on smoking in public areas and selected workplaces, not as much effort has been put into encouraging the adoption of smoke-free rules in private settings.

The aim of the study was to examine involuntary smoking exposure, awareness of its negative health effects, and the factors associated with attitudes towards smoking around young people at home, in vehicles and in the presence of non-smokers among adolescents from a socially disadvantaged rural area in central Poland.

## 2. Material and Methods

### 2.1. Study Design and Population

A cross-sectional study was conducted between November 2014 and May 2015 among secondary and high school students (aged 13–19 years) from the Piotrkowski district, a socially disadvantaged rural area in central Poland (Lodzkie voivodeship—an administrative region of central Poland). A detailed description of the district and the methodology of the study has been published elsewhere [15,16,17,18].

In total, 2997 students attending all 16 secondary schools and 1053 students attending five high schools in the Piotrkowski district were invited to participate in the study. Based on the response rate and the availability of the questionnaire data for the variables of interest (88.3% in the secondary and 86.1% in high schools), the final population covered by the analysis amounted to 3552 students.

In 2014 the study was positively reviewed by the Bioethics Committee of Medical University in Lodz (decision number: RNN/730/14/KB). A written informed consent was received from all the participants or their parents/legal guardians (for underage respondents).

### 2.2. Questionnaire

An anonymous, self-administered questionnaire adopted from the Global Youth Tobacco Survey (GYTS) was completed by the students during regular class hours [19]. The questionnaire consisted of 84 questions (including core questions from the GYTS, as well as country specific questions).

Attitudes towards involuntary smoking exposure were measured with three separate questions: (1) “Should smoking be banned around children at home?”; (2) “Should smoking be banned in vehicles?” and (3) “Should smoking be banned everywhere in the presence of non-smokers?” with three possible answers, i.e.,: “yes”, “no” and “I don’t know”. In the analysis, the responses “no” and “I don’t know” were both considered negative (“no”).

Predictor variables included the following socio-demographic data: gender, school grade (secondary, high), and parental educational level (low: ≤9, medium: 9–12, high: ≥12 years of education). Smoking status of the students was measured with the following question: “Have you ever smoked cigarettes (even a puff)?”. Those who responded “no” were considered “never smokers”. The students who responded “yes” were additionally asked about their current smoking status, which was defined as “smoking within 30 days preceding the study”. The question about parental smoking status was asked separately and in the analysis the following categories were created: “non-smoking parents”, “one or both smoking parents”. The students were also asked about smoking rules at their homes (smoking ban at home: “yes”, “no”), and the length (over the past 7 days) of cohabitation with someone who smoked cigarettes (at home and outside of it). Finally the young respondents reported: (1) their perception of health consequences of active and passive smoking; (2) if they have discussed it with their parents or teachers; and (3) their awareness of the existence of any tobacco control measures in their schools.

### 2.3. Statistical Analysis

The data set is provided in the Supplementary Materials (Table S1). The data are presented as numbers and percentages. Univariable and multivariable logistic regression analyses with the results being presented as odds ratio (OR) and 95% confidence interval (95% CI) were run to study the factors associated with the attitudes of the young people towards smoking around children or adolescents at home, in vehicles, and in the presence of non-smokers. Variables with the *p* value equal to or less than 0.1 from the univariable analysis were included in the multivariable one. A *p* value of less than 0.05 was considered statistically significant. The Statistica Windows XP version 10.0 program (StatSoft Poland Inc., Tulsa, OK, USA) was used to carry out the statistical analysis.

## 3. Results

### 3.1. Characteristics of the Study Population

The descriptive statistics of the population is presented in Table 1. The boys represented 56.4% of the study sample. Most of the young people (74.5%) were attending secondary schools. About 60% of the students indicated that they had ever smoked cigarettes and about 30% that they were current smokers (significantly more boys than girls indicated an ever and current smoking status; *p* < 0.05).

### 3.2. Exposure to Involuntary Smoking among Young People

Almost half of the study participants (48%) reported that at least one of their parent smoked cigarettes, and 60.5% of the respondents indicated they lived at home where smoking was not completely banned (Table 1). In addition, among the group of the young people who indicated a smoking ban at home, 17.3% declared violation of such rules. Nearly 40% of the students who participated in the study were exposed to involuntary smoking at home on a daily, or almost daily basis, and 60% of them outside of it. Significantly higher prevalence of exposure to passive smoking was found in the group of ever-smokers than among the never-smokers.

About 89% of the students were aware of the negative health consequences of active smoking, and 69.3% thought that passive smoking was harmful to health. One third of the study population indicated that they had not discussed smoking-related issues with their family members, and 42.3%—during school classes (Table 1).

### 3.3. Factors Associated with the Attitudes of Young Prople towards Restricting Smoking in the Presence of Non-Smokers

More than 80% of the students felt that smoking should be banned around children at home (Table 1). The percentages dropped to 59.3% in relation to the smoking ban in vehicles and 41.4% in relation to their attitudes towards the smoking ban everywhere in the presence of non-smokers.

Results of the univariable logistic regression analysis evaluating the factors associated with attitudes of young people towards smoking around children and adolescents at home, in vehicles and in the presence of non-smokers have been presented in Table 2.

In the multivariable logistic regression analysis the females were more likely to declare that smoking should be banned around children at home (OR = 1.51; 95% CI 1.22–1.86) and in vehicles (OR = 1.21; 95% CI 1.04–1.41) (Table 3). The youngsters who had never smoked cigarettes and the current non-smokers supported all the three analyzed restrictions regarding exposure to involuntary smoking (around children at home, in vehicles, everywhere in the presence of non-smokers) more frequently than the smokers (*p* < 0.05). Adolescents with no smoking parents, compared to those with at least one smoking parent, were more likely to report that smoking should not be allowed around children at home (OR = 1.44; 95% CI 1.14–1.81), and they thought that this should also be banned in vehicles (OR = 1.27; 95% CI 1.07–1.51). Similarly, those who declared a smoking ban at home supported the smoking ban in vehicles and everywhere in the presence of non-smokers (OR = 1.78; 95% CI 1.51–2.10, OR = 1.59; 95 CI 1.36–1.86 respectively). However, inconsistent results were observed for the question concerning the number of days of exposure to passive smoking at home and outside of it.

The young people who felt that active and passive smoking was harmful to health and who recognized any school tobacco control policies were more likely to report that involuntary smoking should be banned compared to those who were not aware of the health consequences of smoking and any school policies prohibiting tobacco smoking in the school building and around its premises (*p* < 0.05). In addition, information on the health consequences of smoking provided by the parents had a positive impact on the young people’s opinion that smoking should not be allowed around children at home (OR = 1.63; 95% CI 1.32–2.01). Interestingly, the same information shared by a teacher had an even stronger influence (specific education delivered by a teacher resulted in the students supporting all three restrictions regarding exposure to passive smoking; *p* < 0.05).

## 4. Discussion

The current study demonstrates that about 90% of the participants were aware of the health consequences of active smoking, and about 70% of them understood the threats posed by passive smoking. What is more, the majority of the students felt that smoking should not be allowed around children at home. A smaller percentage of them supported a smoking ban in vehicles and everywhere in the presence of non-smokers. It was further observed that despite such awareness, many of the students were still exposed to involuntary smoking in their environment.

A high proportion of young people from a socially disadvantaged rural area in Poland were exposed to involuntary smoking. Almost 50% of all the study population (41% of the never-smokers and 53% of the ever-smokers) had at least one parent who smoked. This coincides with the data collected for the Global Adult Tobacco Survey (GATS) in Poland between 2009 and 2010, indicating that daily and occasional smokers represent 27.8% of the adult population in Polish rural areas [14]. Comparable results were observed in the analysis conducted among never-smoking youth in 168 countries, where parental smoking was indicated by 37% of the respondents [9]. A more detailed analysis based on the GYTS data has indicated that students in the Western Pacific and European Regions were more likely to have one or more smoking parents and those from the African region who indicated parental smoking less frequently [20]. Based on this data, about 59% of young people in Poland in 2003 had at least one parent who smoked, which was more than the number observed in the current study [20]. The differences might be due to many reasons, including: the timing of the study, population characteristics, implementation of tobacco control policies, as well as interventional activities.

Taking into account the fact that parental smoking does not necessarily indicate passive exposure of the adolescents (as they can refrain from smoking in the presence of their children), a smoking ban at home (and its enforcement) seems to be a better estimation of that exposure. In the study, 60% of the respondents indicated they lived at home where smoking was not completely banned (51% of the never-smokers and 67% of the ever-smokers), and close to 40% of the students were exposed to involuntary smoking at their home within the past week (32% of the never-smokers and 44% of the ever-smokers). Similar results related to passive smoking exposure at home were observed among never-smoking adolescents in the European region (54%) [9]. However, these percentages were much lower in a study performed in Canada (2008) where 27% of the students declared that they lived at home where smoking was not completely restricted and that 22% of them were exposed to smoking at their home on a daily or almost daily basis [12].

As in this study, in an analysis carried out in Canada (91% in 2004, 88% in 2006 and 96% in 2008) the majority of young people reported that smoking should not be allowed around children at home [10,11,12]. However, significantly smaller percentages of the population in the current study claimed that smoking should be banned in vehicles (59% vs. 90% in Canada in 2004, 88% in Canada in 2006, and 95% in 2008).

Most likely, the variances result from the different questions used to measure this view. In the study performed in Canada, the students were asked if “smoking should not be allowed around children in vehicles”, whereas in Poland the question was if “it should not be allowed in vehicles”, without the indication of exposure to children. Another reason for the different results can be related to the tobacco control policies, their awareness and enforcement. In Poland, the existing legislation is only restricting smoking in public places (which are still not 100% smoke-free), whereas exposure at home and in vehicles is not regulated [21,22,23]. The federal government in Canada has implemented a program which is designed to help families make their homes and cars smoke-free [10,11,12,24]. In addition, the authorities have enacted legislation prohibiting smoking in vehicles when carrying children [12]. The analysis of the effectiveness of the “Second Hand Smoke in the Home and Car Campaign” indicated that following the program the respondents more frequently reported talking or planning to take action to reduce involuntary smoking exposure [12,25]. Additionally, the study observed that fewer participants had misconceptions about the ways in which to reduce involuntary smoking at home.

The positive outcome of this study is that the majority of the study population was aware of the health consequences of active smoking; however, the awareness of the harmful effects of passive smoking still requires improvement. Based on the analysis among never-smoking youngsters in 168 countries, approximately 90% were aware of the harmful effects of active smoking and passive smoking [9].

The current analysis indicated that the females, never-smokers and current non-smokers, as well as those with no smoking parents were more likely to hold that smoking should be banned at home and in vehicles. This seems to be country, or even region-specific, as the Canadian study demonstrated that males and those exposed to involuntary smoking thought that smoking at home should not be allowed [10,11,12]. The current analysis further confirms that adolescents who were aware of the fact that smoking was harmful to health, who discussed those issues with their parents and teachers, and who recognized any tobacco control policies at school were more likely to report that exposure to passive smoking should be banned. All in all, the results highlighted that educational activities are crucial in addition to the legislation restricting smoking among young people in private spaces (homes and vehicles).

## 5. Study Strengths and Limitations of the Study

The current study has several strengths [15,16,17,18]. Firstly, the study protocol and questionnaire are based on the GYTS standards, which enables a comparison between populations and a time-trend analysis. We used the same definitions of smoking status (ever-smokers, current smokers), involuntary smoking (parental smoking status, smoking ban at home, involuntary smoking during the past week), harmful effects of smoking (for active and passive smoking), and attitudes towards the smoking ban (at home, in vehicles, and in all places where non-smokers would be exposed) as it is determined in the existing studies in the field [9,10,11,12]. Secondly, the data used in the study are based on a large sample size. Finally, the study describes the situation among a disadvantaged population of a rural area, which is usually poorly covered with surveillance. This assures generalizability of the results to other rural areas. Nevertheless, its application to urban regions or other populations can be limited.

The study has also some limitations that merit discussion. The school-based survey included the adolescents who were at the age of selected school attendance, attended school on the day when the survey was administered, and agreed to participate in the study. Based on the definition, adolescents should be considered a population aged 10–19 years [9,26]. The investigators studied young people aged 13–19, so the youngest ones were not included, which might have led to the underestimation of the results (considering the time spent at home). As this study has a cross-sectional design, the causal inferences cannot be established.

Another important limitation of the study is related to the questionnaire data, especially to the questions related to exposure to involuntary smoking in vehicles and everywhere in the presence of non-smokers. As already mentioned above, the questions did not take into account the exposure of children, which can make the results difficult to compare directly with those obtained in other studies. It is vital that future studies separate these issues (investigate beliefs regarding involuntary smoking in general and among children and adolescents). It also needs to be underlined that an assessment of involuntary smoking outside of home is much more difficult and less precise than an assessment of exposure at home. This would explain some inconsistency in the results regarding the beliefs on the smoking ban in the three analyzed situations.

The analysis presented in the paper does not allow for the differentiation of the gender of a smoking parent, nor for an evaluation if it has any impact on the students’ smoking status, involuntary smoking, and their beliefs on the smoking ban in different locations.

Another thing is that active and passive smoking were evaluated by self-reported questionnaire data, which can be related to a recall bias. Although biomarkers of exposure exist (nicotine and cotinine), the cut-off point defined for passive smoking is less clear and more controversial than the cut-off point for active smoking [27]. In addition, the measure of smoking status using biomarkers of exposure in this type of studies (large number of participants) is not always feasible. However, it would be recommended in future studies that such assessments on a subsample population are carried out to verify the information provided by study participants.

Yet another potential limitation is that one cannot be sure of how adolescent or parental smoking is related to episodic or ongoing mental illness, because such information was not collected in the study. Mental health is a strong predictor of smoking and may be associated with smoking patterns [28,29]. In the countries with existing advanced public health campaigns focused on smoking cessation, reduction in the smoking uptake and increased quitting attempts have been observed among general populations. At the same time, in relation to the part of population experiencing common mental health disorders, such as stress, anxiety, and depression, elevated uptake and persistence of smoking remain unchanged. The issue should be addressed in future research.

## 6. Conclusions

Despite the well-known health consequences of active and passive smoking, lack of a safe level of exposure, as well as policies and educational/interventional activities, the current study has demonstrated that a high proportion of young people from a socially disadvantaged rural area in Poland was exposed to involuntary smoking inside and outside of their homes. On the other hand, the majority of the students were aware of the health consequences of smoking and felt that smoking should not be allowed around children at home. The study also highlighted the factors which are associated with positive attitudes towards restricting smoking in the locations where people would be exposed. These findings have implications for smoke-free policies in both public and private venues. Among the school-based programs, especially these focusing not only on prevention of initiation of smoking and its cessation but also addressing protection against passive smoking, are one of the most important. The programs should include education about the health consequences of passive smoking, as well as information on the social and health benefits of smoke-free environments, and the strategies required to make homes and vehicles smoke-free (including avoidance of exposure and the impact of the related parents’ beliefs).

## Figures and Tables

**Table 1:** Descriptive statistics for the study population (N = 3552).

Characteristics	N (%)	Male	Female	Never Smoker	Ever Smoker
		N (%)			
Gender		-	-		
Male	2004 (56.4)			735 (51.6)	1269 (59.7) **^b^**
Female	1548 (46.6)			690 (48.4)	858 (40.3)
School grade					
Secondary	2645 (74.5)	1406 (70.2)	1239 (80.0) **^a^**	1174 (82.4)	1471 (69.2) **^b^**
High	907 (25.5)	598 (29.8)	309 (20.0)	251 (17.6)	656 (30.8)
Mother’s education					
Low	1741 (49.0)	967 (48.2)	774 (50.0)	619 (43.4)	1122 (52.7) **^b^**
Medium	994 (28.0)	588 (29.3)	406 (26.2) **^a^**	313 (22.0)	681 (32.0) **^b^**
High	817 (23.0)	449 (22.4)	368 (23.8)	493 (34.6)	324 (15.2) **^b^**
Father’s education					
Low	2183 (61.5)	1323 (66.0)	860 (55.6) **^a^**	742 (52.1)	1441 (67.7) **^b^**
Medium	786 (22.1)	381 (19.0)	405 (26.2) **^a^**	412 (28.9)	374 (17.6) **^b^**
High	583 (16.4)	300 (15.0)	283 (18.3) **^a^**	272 (19.0)	312 (14.7) **^b^**
Have you ever smoked cigarettes?					
Yes	2127 (59.9)	1269 (63.3)	858 (55.4) **^a^**		
No	1425 (40.1)	735 (36.7)	690 (44.6)		
Current smoking					
Yes	1044 (29.4)	643 (32.1)	401 (25.9) **^a^**		
No	2508 (70.6)	1361 (67.9)	1147 (74.1)		
Parental smoking					
None	1847 (52.0)	1059 (52.8)	788 (50.9)	843 (59.2)	1004 (47.2) **^b^**
One or both parents	1705 (48.0)	945 (47.2)	760 (49.1)	582 (40.8)	1123 (52.8)
Smoking ban at home					
Yes	1404 (39.5)	763 (38.1)	641 (41.4) **^a^**	696 (48.8)	708 (33.3) **^b^**
No	2148 (60.5)	1241 (61.9)	907 (58.6)	729 (51.2)	1419 (66.7)
Enforcement of a smoking ban at home (among those who indicated a smoking ban at home)					
No	243 (17.3)	118 (15.5)	125 (19.5) **^a^**	83 (11.9)	160 (22.6) **^b^**
Yes	1161 (82.7)	645 (84.5)	516 (80.5)	613 (88.1)	548 (77.4)
Over the past 7 days, how many days did you spend at home with someone who smoked cigarettes?					
0 days	2169 (61.1)	1265 (63.1)	904 (58.4) **^a^**	969 (68.0)	1200 (56.4) **^b^**
1–2	537 (15.1)	237 (11.8)	300 (19.4) **^a^**	202 (14.2)	335 (15.7)
3–4	184 (5.2)	141 (7.0)	43 (2.8) **^a^**	67 (4.7)	117 (5.5)
5–6	114 (3.2)	71 (3.5)	43 (2.8)	28 (2.0)	86 (4.0) **^b^**
7 days	548 (15.4)	290 (14.5)	258 (16.7)	159 (11.2)	389 (18.3) **^b^**
Over the past 7 days, how many days did you spend outside of home with someone who smoked cigarettes?					
0 days	1436 (40.4)	731 (36.5)	705 (45.5) **^a^**	873 (61.3)	563 (26.5) **^b^**
1–2	619 (17.4)	391 (19.5)	228 (14.7) **^a^**	270 (18.9)	349 (16.4) **^b^**
3–4	572 (16.1)	412 (20.6)	160 (10.3) **^a^**	182 (12.8)	390 (18.3) **^b^**
5–6	287 (8.1)	173 (8.6)	114 (7.4)	49 (3.4)	238 (11.2) **^b^**
7days	638 (18.0)	297 (14.8)	341 (22.0) **^a^**	51 (3.6)	587 (27.6) **^b^**
Smoking should be banned around children at home					
Yes	2890 (81.4)	1583 (79.0)	1307 (84.4) **^a^**	1288 (90.4)	1602 (75.3) **^b^**
No or I don’t know	662 (18.6)	421 (21.0)	241 (15.5)	137 (9.6)	525 (24.7)
Smoking should be banned in cars					
Yes	2105 (59.3)	1130 (56.4)	975 (653.0) **^a^**	1075 (75.4)	1030 (48.6) **^b^**
No or I don’t know	1447 (40.7)	874 (43.6)	573 (37.0)	350 (24.6)	1097 (51.6)
Smoking should be banned everywhere in the presence of non-smokers					
Yes	1470 (41.4)	847 (42.3)	623 (40.3)	765 (53.7)	705 (33.2) **^b^**
No or I don’t know	2082 (58.6)	1157 (57.1)	925 (59.7)	660 (46.3)	1422 (66.8)
Has someone from your family talked to you about the health consequences of smoking?					
No	1086 (30.6)	479 (23.9)	607 (39.2) **^a^**	466 (32.7)	620 (29.1) **^b^**
Yes	2466 (69.4)	1525 (76.1)	941 (60.8)	959 (67.3)	1507 (70.9)
Perception that tobacco smoking is harmful to health					
No	394 (11.1)	222 (11.1)	172 (11.1)	108 (7.6)	286 (13.4) **^b^**
Yes	3158 (88.9)	1782 (88.9)	1376 (88.9)	1317 (92.4)	1841 (86.6)
Perception that involuntary smoking is harmful to health					
No	1090 (30.7)	594 (29.6)	496 (32.0)	305 (21.4)	785 (36.9) **^b^**
Yes	2462 (69.3)	1410 (70.4)	1052 (68.0)	1120 (78.6)	1342 (63.1)
School policy prohibiting tobacco use within the school building and its premises					
No	1380 (38.9)	776 (38.7)	604 (39.0)	368 (25.8)	1012 (47.6) **^b^**
Yes	2172 (61.1)	1228 (61.3)	944 (61.0)	1057 (74.2)	1115 (52.4)
Have you discussed the health consequences of smoking during school classes?					
No	1503 (42.3)	793 (39.6)	710 (45.9) **^a^**	507 (35.6)	996 (45.8) **^b^**
Yes	2049 (57.7)	1211 (60.4)	838 (54.1)	918 (64.4)	1131 (53.2)

Notes: **^a^** statistically significant differences between males and females (significance level *p* = 0.05); **^b^** statistically significant differences between never smokers and ever smokers (significance level *p* = 0.05).

**Table 2:** Factors associated with the attitudes of young people towards restricting smoking in the presence of non-smokers—unadjusted.

Characteristics	Smoking Should Be Banned around Children	Smoking Should Be Banned in Vehicles	Smoking Should Be Banned Everywhere in the Presence of Non-Smokers
	Unadjusted Odds Ratio (95% CI) N = 3552		
Gender			
Male	1	1	1
Female	1.44 (1.21–1.72) **^a^**	1.31 (1.15–1.51) **^a^**	0.92 (0.80–1.05)
School grade			
Secondary	1.16 (0.96–1.41)	1.47 (1.26–1.71) **^a^**	0.78 (0.67–0.91) **^a^**
High	1	1	1
Have you ever smoked cigarettes?			
Yes	1	1	1
No	3.08 (2.52–3.77) **^a^**	3.27 (2.82–3.78) **^a^**	2.34 (2.04–2.68) **^a^**
Current smoking			
Yes	1	1	1
No	2.17 (1.83–2.59) **^a^**	3.21 (2.76–3.72) **^a^**	2.85 (2.42–3.45) **^a^**
Mother’s education			
Low	1	1	1
Medium	0.88 (0.73–1.07)	1.14 (0.97–1.33)	1.32 (1.13–1.55) **^a^**
High	1.44 (1.14–1.81) **^a^**	1.54 (1.30–1.83) **^a^**	1.88 (1.59–2.23) **^a^**
Father’s education			
Low	1	1	1
Medium	1.48 (1.18–1.84) **^a^**	1.56 (1.32–1.85) **^a^**	1.45 (1.23–1.71) **^a^**
High	1.48 (1.15–1.90) **^a^**	1.13 (0.94–1.37)	1.47 (1.22–1.76) **^a^**
Parental smoking			
Non	1.68 (1.41–1.99) **^a^**	1.65 (1.44–1.88) **^a^**	1.66 (1.45–1.90) **^a^**
One or both parents	1	1	1
Smoking ban at home			
Yes	1.89 (1.58–2.28) **^a^**	2.31 (2. 00–2.67) **^a^**	2.15 (1.88–2.47) **^a^**
No	1	1	1
Enforcement of a smoking ban at home (among these who indicated a smoking ban at home)			
No	1	1	1
Yes	8.20 (5.85–11.49) **^a^**	4.02 (3.01–5.36) **^a^**	3.44 (2.54–4.44) **^a^**
Has someone from your family talked to you the about health consequences of smoking?			
No	1	1	1
Yes	1.76 (1.48–2.10) **^a^**	1.08 (0.94–1.25)	1.10 (0.95–1.27)
Perception that tobacco smoking is harmful to health			
No	1	1	1
Yes	7.01 (5.60–8.77) **^a^**	2.99 (2.40–3.72) **^a^**	2.58 (2.02–3.30) **^a^**
Perception that involuntary smoking is harmful to health			
No	1	1	1
Yes	8.17 (6.72–9.95) **^a^**	2.72 (2.26–3.27) **^a^**	2.28 (1.86–2.78) **^a^**
Over the past 7 days, how many days did you spend at home with someone who smoked cigarettes?			
0 days	1	1	1
1 or more days	0.77 (0.65–0.91) **^a^**	0.75 (0.65–0.85) **^a^**	0.42 (0.37–0.49) **^a^**
Over the past 7 days, how many days did you spend outside of home with someone who smoked cigarettes?			
0 days	1	1	1
1 or more days	1.48 (1.25–1.75) **^a^**	0.52 (0.45–0.60) **^a^**	0.74 (0.65–0.85) **^a^**
Have you discussed the health consequences of smoking during school classes?			
No	1	1	1
Yes	3.19 (2.67–3.81) **^a^**	1.98 (1.73–2.67) **^a^**	1.63 (1.42–1.87) **^a^**
School policy prohibiting tobacco use within the school building and its premises			
No	1	1	1
Yes	3.55(2.97–4.23) **^a^**	3.06 (2.66–3.52) **^a^**	1.81 (1.57–2.08) **^a^**

Note: **^a^** odds ratios statistically significant at the significance level of *p* = 0.05.

**Table 3:** Factors associated with the attitudes of young people towards restricting smoking in the presence of non-smokers—adjusted.

Characteristics	Smoking Should Be Banned around Children at Home	Smoking Should Be Banned in Vehicles	Smoking Should Be Banned Everywhere in the Presence of Non-Smokers
	Adjusted Odds Ratio (95% CI) N = 3552		
Gender			
Male	1	1	
Female	1.51 (1.22–1.86) **^a^**	1.21 (1.04–1.41) **^a^**	*
School grade			
Secondary		0.94 (0.79–1.11)	0.52 (0.44–0.62) **^a^**
High	*	1	1
Mother’s education			
Low	1	1	1
Medium	1.06 (0.84–1.33)	1.26 (1.05–1.50) **^a^**	1.35 (1.15–1.64) **^a^**
High	1.04 (0.76–1.42)	1.03 (0.84–1.26)	1.18 (0.96–1.45)
Father’s education			
Low	1	1	1
Medium	1.20 (0.92–1.57)	1.27 (1.07–1.51) **^a^**	1.24 (1.03–1.49) **^a^**
High	1.18 (0.85–1.64)	1.00 (0.94–1.06)	1.29 (1.04–1.62) **^a^**
Have you ever smoked cigarettes?			
Yes	1	1	1
No	2.08 (1.60–2.72) **^a^**	1.62 (1.34–1.96) **^a^**	1.49 (1.25–1.78) **^a^**
Current smoking			
Yes	1	1	1
No	1.34 (1.05–1.71) **^a^**	1.81 (1.50–2.19) **^a^**	1.99 (1.62–2.43) **^a^**
Parental smoking			
Non	1.44 (1.14–1.81) **^a^**	1.27 (1.07–1.51) **^a^**	0.89 (0.75–1.05)
One or both parents	1	1	1
Smoking ban at home			
Yes	1.02 (0.79–1.31)	1.78 (1.51–2.10) **^a^**	1.59 (1.36–1.86) **^a^**
No	1	1	1
Over the past 7 days, how many days did you spend at home with someone who smoked cigarettes?			
0 days	1	1	1
1 or more days	1.19 (0.93–1.51)	1.47 (1.22–1.75) **^a^**	0.54 (0.45–0.65) **^a^**
Over the past 7 days, how many days did you spend outside of home with someone who smoked cigarettes?			
0 days	1	1	1
1 or more days	1.82 (1.44–2.29) **^a^**	0.65 (0.55–0.79) **^a^**	1.05 (0.89–1.24)
Has someone from your family talked to you about the health consequences of smoking?			
No	1		
Yes	1.63 (1.32–2.01) **^a^**	*	*
Perception that tobacco smoking is harmful to health			
No	1	1	1
Yes	3.51 (2.68–4.59) **^a^**	2.05 (1.59–2.62) **^a^**	1.81 (1.38–2.37) **^a^**
Perception that involuntary smoking is harmful to health			
No	1	1	1
Yes	3.87 (3.08–4.86) **^a^**	1.54 (1.24–1.91) **^a^**	1.44 (1.15–1.81) **^a^**
Have you discussed the health consequences of smoking during school classes?			
No	1	1	1
Yes	2.19 (1.77–2.71) **^a^**	1.51 (1.29–1.78) **^a^**	1.35 (1.16–1.59) **^a^**
School policy prohibiting tobacco within the school building and its premises			
No	1	1	1
Yes	1.94 (1.57–2.39) **^a^**	2.03 (1.73–2.38) **^a^**	1.31 (1.11–1.54) **^a^**

Note: * Variables with p value higher than 0.1 from the univariable analysis were not included in the multivariable analysis. **^a^** odds ratios statistically significant at the significance level of *p* = 0.05.

## Supplementary Materials

The following are available online at www.mdpi.com/1660-4601/14/10/1095/s1, Table S1: Data set.
